# Microbial community assembly along a groundwater–hypersaline lake continuum in the Badain Jaran Desert

**DOI:** 10.1093/femsec/fiag113

**Published:** 2026-10-05

**Authors:** Meiqing Lu, Xiaolang Zhang, Jiu Jimmy Jiao, Jinchao Zuo, Hailong Li, Meng Li

**Affiliations:** CUG-HKU Joint Centre for Coastal Groundwater and Ecosystem Studies, Department of Earth and Planetary Sciences, The University of Hong Kong, Hong Kong SAR, China; Archaeal Biology Centre, Synthetic Biology Research Center, Shenzhen Key Laboratory of Marine Microbiome Engineering, Key Laboratory of Marine Microbiome Engineering of Guangdong Higher Education Institutes, Institute for Advanced Study, Shenzhen University, Shenzhen 518060, China; Shenzhen Institute of Research and Innovation, The University of Hong Kong, Shenzhen 518057, China; Department of Geosciences, Florida Atlantic University, Boca Raton, FL 33431, United States; CUG-HKU Joint Centre for Coastal Groundwater and Ecosystem Studies, Department of Earth and Planetary Sciences, The University of Hong Kong, Hong Kong SAR, China; Shenzhen Institute of Research and Innovation, The University of Hong Kong, Shenzhen 518057, China; CUG-HKU Joint Centre for Coastal Groundwater and Ecosystem Studies, Department of Earth and Planetary Sciences, The University of Hong Kong, Hong Kong SAR, China; State Key Laboratory of Soil Pollution Control and Safety, Southern University of Science and Technology, Shenzhen 518055, China; Archaeal Biology Centre, Synthetic Biology Research Center, Shenzhen Key Laboratory of Marine Microbiome Engineering, Key Laboratory of Marine Microbiome Engineering of Guangdong Higher Education Institutes, Institute for Advanced Study, Shenzhen University, Shenzhen 518060, China

**Keywords:** groundwater-fed hypersaline lake, community assembly, salinity gradient, Badain Jaran Desert, terminal lake

## Abstract

Microbial communities along groundwater–hypersaline lake continua encounter steep physicochemical gradients over short distances, yet how hydrological connectivity and geochemical gradients jointly shape community assembly remains poorly understood. Here, we combined hydrogeochemical profiling, 16S rRNA gene amplicon sequencing, indicator species analysis, null-model inference, and predictive functional profiling to characterize community composition, assembly processes, and functional potential across 88 samples at South Sumujaran Lake, Badain Jaran Desert, China. The samples spanned fresh groundwater in two transects, brackish-to-saline groundwater in the transition zone, and hypersaline lake water along a hydrologically connected continuum, covering a more than 300-fold range in salinity. Despite hydrological connectivity, lake water communities were almost completely distinct from those in the two groundwater transects and the transition zone. The transition zone also supported a distinct set of indicator taxa, suggesting that its community composition was not explained by mixing alone. Assembly processes shifted along the continuum: dispersal limitation was the most prevalent process system-wide and occurred within the two low-salinity groundwater transects but not in the transition zone or hypersaline lake, whereas homogeneous selection increasingly structured communities toward these higher-salinity zones. Predicted functional profiles differed little between the two groundwater transects but markedly between groundwater and lake water. This zone-specific pattern helps explain how steep solute gradients structure microbial communities along hydrologically connected flow paths in groundwater-fed terminal lakes of arid regions.

## Teaser

Desert groundwater flows continuously into a salt lake, but its microbial communities stay separate—chemistry, not water movement, controls where each microbe lives.

## Introduction

Groundwater–hypersaline lake continua are hydrologically and biogeochemically coupled systems in which subsurface transport and evaporative concentration connect fresh groundwater with hypersaline lake water (Wang et al. 2014, Zhang et al. 2021a). Such continua are particularly prominent in arid regions, where groundwater discharge sustains terminal lakes under intense evaporation, producing steep salinity gradients over short spatial distances of tens to hundreds of meters (Hammer 1986, Williams 2002). Along these continua, hydrological connectivity, solute transport, and water residence time jointly regulate physicochemical conditions, including salinity, alkalinity, and redox state (Zhang et al. 2021a, Yu et al. 2025). These conditions impose environmental constraints that are expected to shape the composition and functional potential of resident microbial communities.

Microorganisms along these continua encounter contrasting habitats, from oligotrophic fresh groundwater to hypersaline lake water. Salinity is a major driver of microbial community structure, imposing osmotic stress that can reduce diversity and favor specialized taxa at high salinities (Lozupone and Knight 2007, Oren 2011). However, many previous studies have focused on discrete freshwater or hypersaline sites, or on surface-water gradients without explicitly considering subsurface hydrological processes (Casamayor et al. 2002, Lozupone and Knight 2007, Lu et al. 2019). Groundwater-fed terminal lakes offer a contrasting setting in which groundwater flow links subsurface and surface environments (Zhang et al. 2021a, Yu et al. 2025). This connectivity provides opportunities for microbial dispersal, while sharp physicochemical transitions may constrain the establishment of transported taxa. How these two influences jointly shape microbial community assembly along groundwater–hypersaline lake continua remains poorly understood.

Beyond salinity, groundwater flow through heterogeneous sediments can generate redox zonation that is partly decoupled from the primary salinity gradient (McMahon et al. 2011, Kolbe et al. 2019). Organic-rich paleolake deposits may sustain reducing conditions that favor nitrate depletion and phosphorus mobilization (Boano et al. 2014, Kuypers et al. 2018, Shen et al. 2018, Li et al. 2023). Groundwater–surface water transition zones can also act as biogeochemical hotspots, where redox gradients and the availability of organic matter and nutrients support microbial activity (McClain et al. 2003, Lewandowski et al. 2015, Stegen et al. 2016, Fillinger et al. 2019). Sedimentary legacies in the aquifer and biogeochemical hotspots at transition zones may therefore introduce additional environmental filters beyond salinity. Their contribution to community assembly along complete groundwater–hypersaline lake continua, however, remains insufficiently understood.

Groundwater-fed terminal lakes are widespread in arid and semi-arid basins worldwide, and the Badain Jaran Desert in northwestern China provides a natural setting for investigating these processes. This hyper-arid region receives less than 100 mm of precipitation annually and contains more than 100 perennial lakes sustained primarily by groundwater discharge (Chen et al. 2004, Gates et al. 2008, Jiao et al. 2015). South Sumujaran Lake exemplifies the transition from fresh groundwater to hypersaline lake water, accompanied by changes in pH, alkalinity, nutrient stoichiometry, and redox conditions (Zhang et al. 2021a, 2021b). Here, “continuum” denotes hydrological connectivity across a geochemical gradient and does not imply continuity in microbial community composition. Our previous hydrogeochemical study identified two contrasting groundwater settings: an eastern sandy aquifer and a western transect influenced by organic-rich paleolake deposits (Zhang et al. 2021a). The two transects have similarly low salinities but markedly different redox conditions, providing an opportunity to examine whether redox conditions associated with sedimentary legacy contribute to community differentiation beyond the effects of salinity.

Given this setting and the well-documented effect of salinity on microbial diversity, we expected strong differentiation in community composition among hydrogeochemical zones and declining diversity toward the hypersaline endpoint. We further hypothesized that deterministic selection would become more prominent toward the high-salinity zones. Two additional questions concerned the roles of local environmental conditions: whether transition-zone communities simply reflected mixing between adjacent zones or also showed evidence of local environmental selection, and whether redox conditions associated with paleolake deposits in the western transect acted as an additional environmental filter.

To address these hypotheses and questions, we combined hydrogeochemical profiling, 16S rRNA gene amplicon sequencing, indicator species analysis, null-model inference, and predictive functional profiling across 88 samples spanning the groundwater–hypersaline lake continuum of South Sumujaran Lake. We estimated the relative contributions of community assembly processes at two scales: system-wide comparisons (among all 88 samples) and within individual hydrogeochemical zones. By linking community patterns with environmental conditions and assembly processes, this study examines how dispersal and selection jointly structure microbial communities along hydrologically connected flow paths with steep geochemical gradients in groundwater-fed terminal lakes.

## Materials and methods

### Study area

This study was conducted at South Sumujaran Lake and its adjacent groundwater aquifer in the Badain Jaran Desert, northwestern China (39°47′N, 102°25′E), ~50 km from the Yabulai Mountains (Fig. 1a). The lake has a surface area of 1.26 km² and a maximum depth of ~11 m (Zhang et al. 2021a).

**Figure 1:**
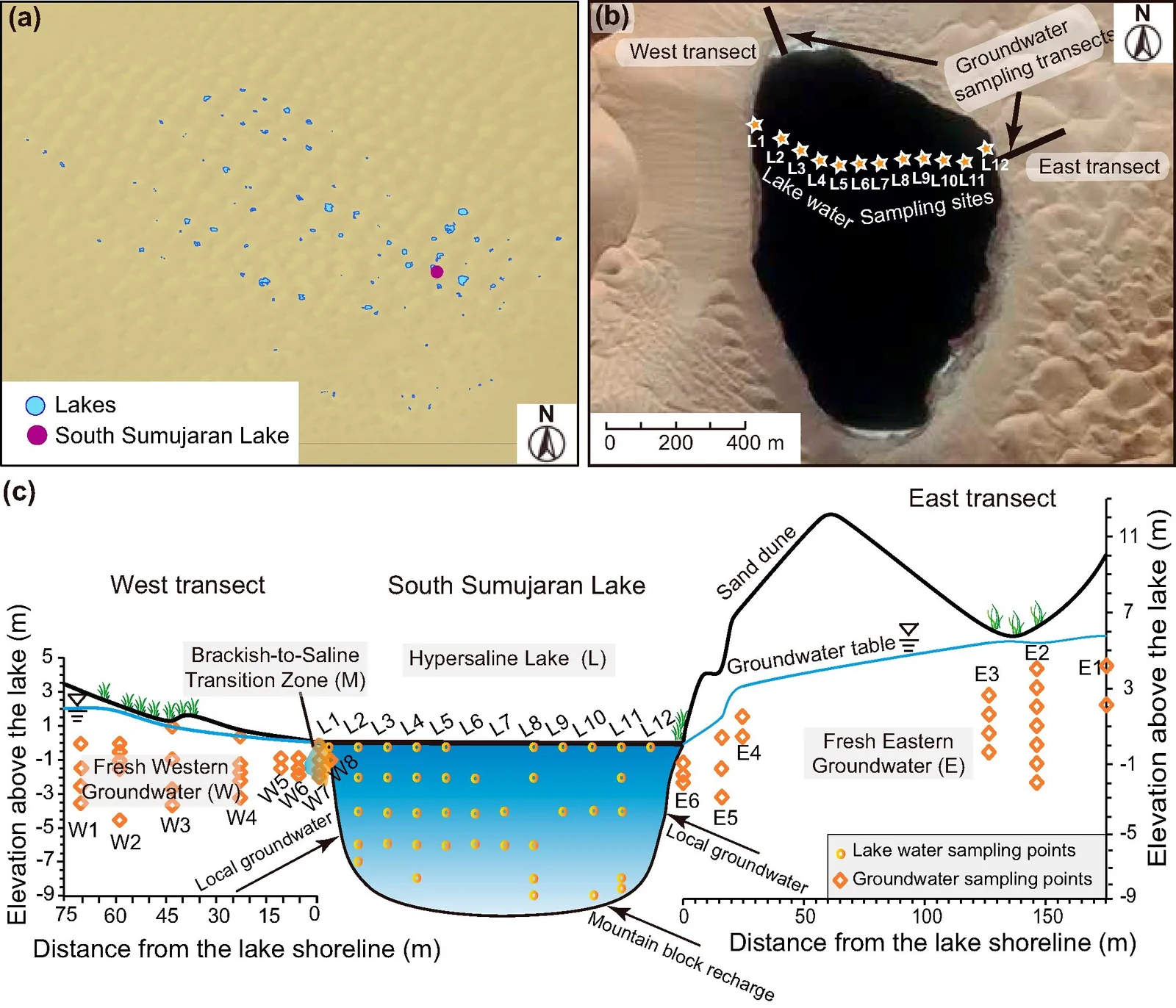
Study site and sampling design. (a) Distribution of lakes in the Badain Jaran Desert, with South Sumujaran Lake highlighted. (b) Satellite image of South Sumujaran Lake showing the lake-water sampling sites (L1–L12) and the two groundwater transects. (c) Cross-section of the groundwater–lake continuum showing the western transect, transition zone, hypersaline lake, and eastern transect, with sampling positions (elevation relative to the lake surface) and groundwater flow; adapted from Zhang et al. (2021a), *Journal of Hydrology*, Copyright (2021), with permission from Elsevier. Zones used throughout: E, fresh eastern groundwater; W, fresh western groundwater; M, brackish-to-saline transition zone; and L, hypersaline lake water.

The Badain Jaran Desert is characterized by a hyper-arid climate, with mean annual precipitation below 100 mm and potential evaporation exceeding precipitation by more than an order of magnitude. Surface runoff is negligible, and perennial lakes are sustained primarily by groundwater discharge from both local flow systems and regional mountain-block recharge (Zhang et al. 2021b).

Four hydrologically and geochemically distinct zones were delineated (Fig. 1b and c): (i) the hypersaline lake, comprising 12 sites (L1–L12; zone L), (ii) fresh eastern groundwater consisting of six multilevel wells (E1–E6; zone E), (iii) fresh western groundwater consisting of wells W1–W6 (zone W), and (iv) a brackish-to-saline transition zone represented by wells W7–W8 at the interface between the western groundwater and the lake (zone M).

The eastern transect is dominated by sandy sediments, whereas the western transect consists of mixed sand and black silt, with silt content increasing toward the lakeshore. This sedimentary contrast reflects the influence of paleolake deposits formed during higher lake-level stages (Zhang et al. 2021a), providing geological context for the hydrological and geochemical variability observed across transects.

### Field sampling

Sampling was conducted in April 2019 to characterize microbial and physicochemical variation across the coupled groundwater–lake system while minimizing temporal variability.

The twelve lake sites were sampled from a motorized raft, with sampling depths adjusted according to local water depth to resolve vertical structure where present, yielding hypersaline lake water (L, *n* = 39). Two multilevel groundwater transects were installed on the eastern and western shores (Fig. 1b and c); each well consisted of multiple nylon tubes with screened inlets at different depths to characterize vertical profiles. The eastern transect (E1–E6) yielded fresh groundwater (E, *n* = 21). The western transect exhibited a pronounced salinity gradient and was subdivided accordingly: wells W1–W6 yielded fresh groundwater (W, *n* = 23), whereas wells W7–W8 yielded brackish-to-saline water (M, *n* = 5).

All samples were collected in sterile containers. For microbial analysis, 1 l of water per sample was filtered through 0.22 μm polycarbonate membrane filters (Isopore GTTP02500, 25 mm; Merck Millipore, Cork, Ireland) immediately after collection, using multiple filters per sample that were pooled for DNA extraction. Filters were transported on dry ice and stored at −80°C until extraction.

### Physicochemical measurements and laboratory analyses

pH, temperature, and oxidation–reduction potential (ORP) were measured immediately after sample collection using a portable multiparameter probe (Hanna HI 98194), with reported accuracies of ±0.02, ±0.15°C, and ±1.0 mV, respectively. Total dissolved solids (TDS; ±1% of reading) were measured with the same probe. Because practical salinity is defined for seawater-type ionic composition and does not apply to these carbonate-dominated brines, salinity is reported as TDS in g l⁻¹ throughout (Hammer 1986, Williams 2002).

Ammonium (NH_4_^+^), nitrite (NO_2_⁻), nitrate (NO_3_⁻), soluble reactive phosphorus (SRP, measured as phosphate), and dissolved silicon (DSi) were determined using a flow injection analyzer (Lachat QuickChem 8000) following standard colorimetric methods, with analytical uncertainties <10% for NH₄⁺, <8% for NO_2_⁻ and NO_3_⁻, and <5% for SRP and DSi. Dissolved organic carbon (DOC) and total nitrogen (TN) concentrations were determined using a TOC/TN analyzer (multi N/C 3000, Analytik Jena AG, Germany), with analytical errors <3%. Analytical procedures followed Zhang et al. (2021a). Alkalinity was determined by titration using a Hach alkalinity test kit (Model AL-AP) and is reported as mg l⁻¹ CaCO_3_. To characterize nutrient structure and biogeochemical gradients along the groundwater–hypersaline lake continuum, three molar ratios were calculated: DSi:SRP, TN:SRP, and DOC:TN.

Radon (^222^Rn) activities were quantified using a RAD7 detector following a gas–water equilibration approach (Burnett et al. 2001). Activities were recorded at 15-min intervals, and chamber temperature was logged concurrently. Radon was used as a tracer of groundwater discharge and groundwater–lake exchange (Shi et al. 2024). Hydrogeochemical structure across samples was summarized by principal component analysis (PCA) of the 17 *z*-scored physicochemical variables, restricted to the 84 samples with complete records.

### DNA extraction, amplicon sequencing, and bioinformatics

DNA was extracted from 0.22 μm filters using the DNeasy PowerWater DNA Isolation Kit (formerly MoBio; Qiagen, Hilden, Germany) following the manufacturer’s protocol. DNA concentration and purity were assessed using a NanoDrop One spectrophotometer (Thermo Fisher Scientific, Waltham, MA, USA).

The V4 region of the 16S rRNA gene was amplified using the modified 515F/806R primer pair (GTGYCAGCMGCCGCGGTAA/GGACTACNVGGGTWTCTAAT) (Apprill et al. 2015, Parada et al. 2016, Walters et al. 2016). Amplicons were purified and sequenced on an Illumina MiSeq platform (2 × 300 bp paired-end), targeting a minimum of 50 000 reads per sample.

Paired-end reads were processed in QIIME 2 v2024.10 (Bolyen et al. 2019). Primers were removed with cutadapt, discarding read pairs without detectable primer sequences. Amplicon sequence variants (ASVs) were inferred with DADA2 (Callahan et al. 2016), with forward and reverse reads truncated at 240 and 220 bp, respectively, based on the quality profiles. The maximum expected-error threshold was set to 2 for both read directions, and all other DADA2 parameters were left at their defaults, including consensus chimera removal. Taxonomy was assigned with q2-feature-classifier using the pre-trained naïve Bayes classifier for SILVA 138 (99% OTUs, full-length sequences) (Quast et al. 2013, Bokulich et al. 2018, Robeson et al. 2021), as distributed with QIIME 2 (artefact UUID: 70b4b5f4-8fce-40bd-b508-afacbc12a5ed).

Sequence processing yielded 22 393 ASVs across 88 samples after removal of ASVs with a total abundance below seven reads (Xiao et al. 2025). Chloroplast-derived (37), mitochondrion-derived (164) and domain-unassigned (134) ASVs were then removed, together accounting for 0.74% of reads. After taxonomic filtering, 22 058 ASVs remained. Rarefaction to 61 007 reads per sample retained all 88 samples and 21 993 ASVs; the 65 ASVs lost had 7–11 reads in total before rarefaction. Community analyses used the rarefied table, whereas PICRUSt2 predictions used the filtered, unrarefied table. The filtered unrarefied and rarefied ASV tables are provided as Supplementary Data 2 and Supplementary Data 3, respectively.

### Community and statistical analyses

Rarefaction curves were used to evaluate sequencing depth sufficiency (Hughes et al. 2001). Alpha diversity was quantified using observed ASV richness and the Shannon index (Shannon 1948). Differences in richness and Shannon diversity among zones were tested by Kruskal–Wallis rank-sum tests. Beta diversity was assessed using Bray–Curtis dissimilarity (Bray and Curtis 1957) and unweighted UniFrac distance (Lozupone and Knight 2005), the latter computed on a phylogenetic tree of all 21 993 ASVs constructed as described below. Within-zone heterogeneity was summarized as the median pairwise dissimilarity among samples of the same zone, using Bray–Curtis dissimilarity on relative abundances, unweighted UniFrac distance, and Jaccard distance on presence–absence data (Jaccard 1912).

Community analyses were performed on the ASV table rarefied to 61 007 reads per sample, with taxonomic composition expressed as relative abundance. Differences in community composition among the four hydrogeochemical zones were tested by analysis of similarities (ANOSIM) on Bray–Curtis dissimilarities with 9999 permutations (Clarke 1993). This was complemented by permutational multivariate analysis of variance (PERMANOVA; adonis2) and by a test of homogeneity of multivariate dispersion (betadisper, distances to group centroids), both with 9 999 permutations. The betadisper test was applied to Bray–Curtis, unweighted UniFrac, and Jaccard distances, with Bonferroni-corrected pairwise comparisons. All analyses were implemented in vegan v2.6–2 (Oksanen et al. 2022). Because multivariate dispersion differed among zones, ANOSIM and PERMANOVA results are interpreted as reflecting a combination of location and dispersion effects. Principal coordinates analysis (PCoA) was used to visualize among-sample dissimilarities (Gower 1966). Differences in the relative abundances of the 10 most abundant bacterial phyla and five most abundant archaeal classes among the four zones were assessed using Kruskal–Wallis rank-sum tests. Where the overall test was significant, pairwise comparisons were conducted using Dunn’s post hoc test with Bonferroni correction, implemented in dunn.test v1.4.1 (Dinno 2026). Because this implementation reports one-sided *P*-values, the Bonferroni-adjusted one-sided *P*-values were multiplied by two so that all reported comparisons correspond to two-sided tests; values exceeding 1 after doubling were treated as non-significant.

Shared and unique ASVs across zones were summarized using UpSet plots (Conway et al. 2017). For indicator analysis, the rarefied ASV table was further normalized by the trimmed mean of *M*-values method in edgeR v3.42.4 (Robinson et al. 2010) and expressed as counts per million. Zone-associated taxa were identified with the multipatt function of indicspecies v1.7.14 (De Cáceres and Legendre 2009), using the group-equalized point-biserial correlation coefficient (*r*_g_) as the association statistic with 9999 permutations. This statistic was chosen because zone sample sizes are markedly unequal, and the group-size correction is designed for this situation. Because multipatt evaluates all combinations of zones, a taxon may be identified as an indicator of a single zone or of a combination of zones. *P*-values were corrected for multiple testing across all tested ASVs using the Benjamini–Hochberg procedure, and taxa were retained as indicators when *r*_g_ > 0.3 and adjusted *P* < 0.05; taxa lacking phylum-level assignment were excluded. A fixed random seed (8046) was used throughout. Indicator taxonomic composition was summarized as the number of indicator ASVs assigned to each phylum within each zone.

Assembly processes were evaluated using a null-model framework to assess the relative contributions of deterministic and stochastic processes (Stegen et al. 2013). The primary analysis included ASVs with a mean relative abundance of at least 1 × 10⁻^4^ across all 88 samples (670 ASVs); sensitivity to this criterion was evaluated using two additional occurrence thresholds. Representative sequences were aligned with MAFFT, and the alignment was masked to remove highly variable positions. An approximately maximum-likelihood phylogenetic tree was then inferred with FastTree and midpoint-rooted using the align-to-tree-mafft-fasttree pipeline in QIIME 2 v2024.10, with default masking parameters (Price et al. 2010, Katoh and Standley 2013). The tree was subsequently pruned to the ASVs retained for the null-model analysis. The pruned tree is provided as Supplementary Data 5 to facilitate reproducibility. The β nearest taxon index (βNTI) was calculated from the abundance-weighted β mean nearest taxon distance (βMNTD; comdistnt, abundance.weighted = TRUE) using picante v1.8.2 and ape v5.7–1, while the abundance-based, Bray–Curtis Raup–Crick metric (RCbray), based on the Raup–Crick null model (Chase et al. 2011), was calculated by assembling null communities in proportion to taxon occurrence frequency and regional relative abundance across the whole dataset, with the resulting probability rescaled to the −1 to +1 interval (Stegen et al. 2013). Both statistics used 999 randomizations under a fixed random seed. Pairwise comparisons with βNTI ≤ −2 were assigned to homogeneous selection and those with βNTI ≥ +2 to variable selection. Where |βNTI| < 2, comparisons were assigned to homogenizing dispersal (RCbray ≤ −0.95), dispersal limitation (RCbray ≥ +0.95), or undominated processes (|RCbray| < 0.95). Contributions are reported as counts alongside percentages and Wilson 95% confidence intervals; because pairwise comparisons share samples and are not independent, these intervals are approximate and are reported for descriptive purposes. Because the absolute contributions inferred from null models depend on how many ASVs are retained, the complete workflow was repeated under two further filtering criteria (presence in at least 10 samples, 949 ASVs; presence in at least five samples, 2 857 ASVs); results under all three criteria are given in Supplementary Data 7. The proportion of within-zone comparisons falling close to the βNTI threshold of −2 was also examined for each zone. Phylogenetic signal in ASV environmental optima was assessed with a Mantel correlogram based on these 670 ASVs. Environmental conditions were summarized as sample scores on the first principal component of the 17 *z*-scored environmental variables (84 samples with complete environmental records), rescaled to the 0–1 interval, and the environmental optimum of each ASV was calculated as the abundance-weighted mean of these scores across samples. Differences in optima between ASVs were related to cophenetic phylogenetic distances in 50 distance classes of width 0.02 using mantel.correlog in vegan (999 permutations, Bonferroni correction), as implemented in microeco v1.7.1 (Liu et al. 2021).

Relationships between community composition and environmental variables were examined by redundancy analysis (RDA) on Hellinger-transformed ASV abundances (Legendre and Gallagher 2001), using vegan v2.6–2 (Oksanen et al. 2022). Samples with incomplete environmental records were excluded, leaving 84 of 88 samples. Because several environmental variables were strongly collinear (salinity and alkalinity, *r* = 0.999; variance inflation factors 574 and 618), variable selection was performed by forward selection on adjusted *R*² using ordiR2step with 999 permutations, rather than by testing all variables simultaneously. The significance of variables retained in the final model was assessed by marginal permutation tests, and the collinearity between salinity and alkalinity was additionally examined by marginal tests in the full 17-variable model. For each environmental variable considered individually, a separate one-variable constrained ordination was fitted, and both the constrained *R*² and adjusted *R*² were calculated using RsquareAdj. The relative influence of salinity- and redox-related variables was assessed by variation partitioning with these variables treated as groups, applied to community composition; salinity, alkalinity, and pH formed the salinity-related group, and ORP, NH₄⁺, and NO₃⁻ the redox-related group. Relationships between pairwise βNTI and environmental variables were assessed by Mantel tests relating the signed βNTI matrix to the absolute between-sample difference in each variable (9999 permutations).

Environmental dissimilarity was computed as Euclidean distance on the 17 *z*-scored (standardized) physicochemical variables. Geographic distances between samples were computed along a common horizontal axis with its origin at the western shoreline, using along-transect distances together with the vertical separation given by relative elevation. Because eastern-transect positions are measured from the eastern shoreline whereas all other positions are measured from the western shoreline, they were offset by a fixed distance. This offset, 1476 m, is the along-transect span between the outermost lake stations, taken as a lower-bound approximation of the lake width. Partial Mantel results were insensitive to this choice: offsets of 1400–1750 m yielded correlations within 0.01. Distance decay was tested by Mantel and partial Mantel tests with 9999 permutations.

Functional profiles were predicted with PICRUSt2 v2.5.2 (Douglas et al. 2020) using the default reference database. Of the 22 058 ASVs submitted, 42 aligned poorly to the reference alignment and 1143 exceeded the nearest-sequenced-taxon index cut-off of 2.0; the remaining 20 873 ASVs (94.6%) yielded 8029 Kyoto Encyclopedia of Genes and Genomes (KEGG) orthologues, which were mapped to KEGG pathway categories using the BRITE hierarchy. Functional profiles were compared among zones by principal coordinate analysis on Bray–Curtis dissimilarities of KO relative abundances and by ANOSIM. MetaCyc pathway abundances were also predicted using the default PICRUSt2 pipeline (Supplementary Data 9). KEGG Level 2 categories were compared between zone pairs by Wilcoxon rank-sum tests with Benjamini–Hochberg correction. Predicted functional profiles are inferences from gene content and do not constitute direct evidence of metabolic activity.

All statistical analyses were performed in R v4.3.1. Statistical significance was assessed at *P* < 0.05. The raw sequence data reported in this paper have been deposited in the Genome Sequence Archive (Zhang et al. 2025) at the National Genomics Data Center (Bao et al. 2025), China National Center for Bioinformation/Beijing Institute of Genomics, Chinese Academy of Sciences (GSA: CRA048363). These data are publicly accessible at https://ngdc.cncb.ac.cn/gsa. The R scripts, input tables and outputs used for the null-model and indicator-species analyses are provided as Supplementary Code.

## Results

### Physicochemical gradients define the groundwater–hypersaline lake continuum

Hydrogeochemical measurements revealed pronounced physicochemical differences among four hydrologically connected zones: fresh eastern groundwater (E), fresh western groundwater (W), the brackish-to-saline transition zone (M), and hypersaline lake water (L) (Supplementary Data 1; Table S1). Unless otherwise stated, values represent zone means.

Salinity, reported here as TDS, increased by more than two orders of magnitude, from 0.49 and 0.40 g l⁻¹ in eastern and western groundwater, respectively, to 18.05 g l⁻¹ in the transition zone and 126.53 g l⁻¹ in lake water. This increase was accompanied by higher pH and alkalinity. Mean pH was 8.10 in eastern groundwater and 8.59 in western groundwater, compared with 10.32 in the transition zone and 10.27 in lake water. Alkalinity increased from 155 and 220 mg l⁻¹ in eastern and western groundwater, respectively, to 7440 mg l⁻¹ in the transition zone and 40,628 mg l⁻¹ in lake water. Despite their similarly low salinities, the two groundwater transects differed markedly in ORP. Mean ORP was positive in eastern groundwater (62.73 mV) but negative in western groundwater (−117.47 mV) and the transition zone (−221.88 mV). Lake-water ORP varied widely, ranging from −224.8 to +74.9 mV, with a mean of 4.75 mV. Mean water temperature ranged from 10.1 to 15.6°C among zones. Mean ²²²Rn activities were highest in eastern groundwater (345.25 dpm l⁻¹), followed by western groundwater (104.63 dpm l⁻¹), the transition zone (68.52 dpm l⁻¹), and lake water (15.50 dpm l⁻¹), consistent with sustained groundwater influence along the continuum.

NH₄⁺ and SRP concentrations peaked in the transition zone. Mean NH₄⁺ concentrations were 10.42 and 10.17 μM in eastern and western groundwater, respectively, increased to 195.33 μM in the transition zone, and declined to 20.29 μM in lake water. SRP showed a similar pattern, with corresponding concentrations of 3.25, 3.96, 43.55, and 25.62 μM. DOC increased from 0.44 mM in eastern groundwater to 6.82 mM in lake water. The transition zone also exhibited the highest mean DOC:TN ratio (179.71) and the lowest mean TN:SRP and DSi:SRP ratios (0.59 and 2.45, respectively), reflecting phosphorus enrichment relative to nitrogen and silicon in this zone.

PCA of the 17 standardized physicochemical variables was based on the 84 samples with complete environmental records. The first two axes together explained 62.0% of the total variance (PC1: 43.4%; PC2: 18.6%; Fig. 2a). PC1 had strong positive loadings for pH, DOC, alkalinity, salinity, and SRP, and negative loadings for DSi:SRP, ²²²Rn, TN:SRP, NO₃⁻, and relative elevation. This axis represented a coupled dissolved-solute, alkalinity, and organic matter gradient separating fresh groundwater from hypersaline lake water (Fig. 2b). PC2 had strong positive loadings for ORP and TN and negative loadings for DOC:TN and NH₄⁺, capturing variation in redox conditions and nitrogen chemistry that distinguished eastern groundwater from western groundwater and the transition zone (Fig. 2b).

**Figure 2:**
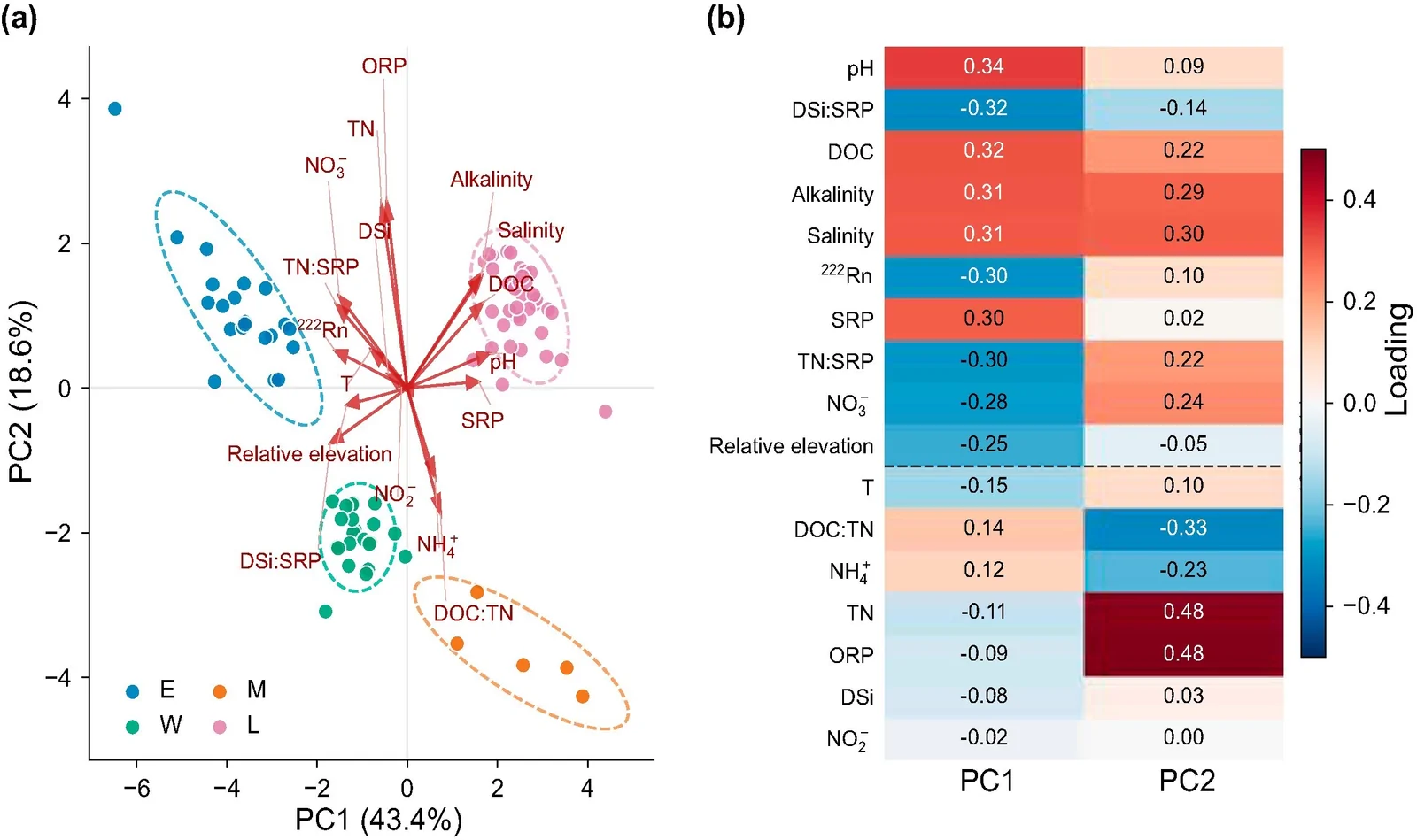
Hydrogeochemical structure across the continuum. (a) PCA of 17 physicochemical variables for the 84 samples with complete records; ellipses are 95% confidence regions. (b) Loadings of individual variables on the first two PCA axes; the dashed line separates the 10 variables with the largest absolute PC1 loadings. DOC, dissolved organic carbon; TN, total nitrogen; SRP, soluble reactive phosphorus; DSi, dissolved silicon; ORP, oxidation–reduction potential; T, temperature; NH_4_⁺, ammonium; NO_2_⁻, nitrite; and NO_3_⁻, nitrate.

### Microbial community composition and diversity patterns

After quality control, taxonomic filtering, and rarefaction to 61 007 reads per sample, 21 993 ASVs were retained, comprising 20 588 bacterial ASVs (93.6%) and 1405 archaeal ASVs (6.4%) (Supplementary Data 2, 3). Before rarefaction, sequencing depths ranged from 61 007 to 172 090 reads per sample, and rarefaction curves plateaued before the selected rarefaction depth (Fig. S1).

Bacterial communities were dominated by *Pseudomonadota* (formerly *Proteobacteria*; 51.45% ± 13.85% of rarefied reads), followed by *Bacteroidota* (formerly *Bacteroidetes*; 11.55% ± 6.36%), *Bacillota* (formerly *Firmicutes*; 11.21% ± 9.35%), *Actinomycetota* (formerly *Actinobacteriota*; 8.17% ± 8.14%), and *Verrucomicrobiota* (3.34% ± 4.52%) (Fig. 3a; Supplementary Data 4) (Oren and Garrity 2021). Bacterial and archaeal taxa showed distinct patterns of relative abundance among zones (Fig. S2). *Nitrososphaeria, Iainarchaeia, Acidobacteriota, Bdellovibrionota*, and *Verrucomicrobiota* were significantly more abundant in both fresh groundwater transects than in lake water, and *Bdellovibrionota* was also more abundant in eastern than in western groundwater. *Bathyarchaeia* and *Desulfobacterota* were enriched in western groundwater and the transition zone relative to eastern groundwater and lake water. *Halobacteria, Actinomycetota*, and *Bacillota* were significantly more abundant in lake water than in either fresh groundwater transect, although *Bacillota* reached its highest relative abundance in the transition zone. In contrast, *Nanoarchaeia, Chloroflexota*, and *Patescibacteria* were significantly less abundant in lake water than in the other three zones.

**Figure 3:**
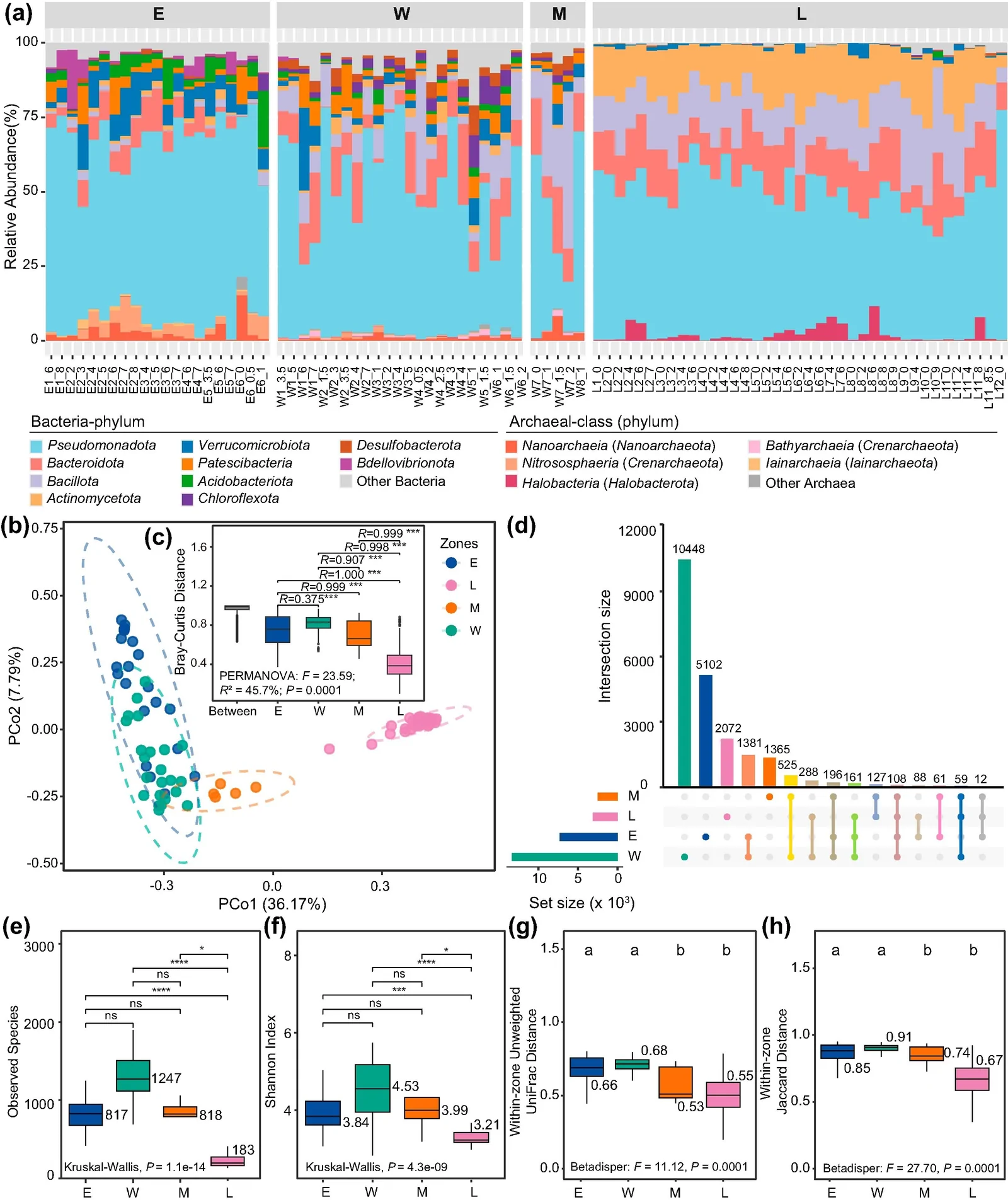
Microbial community composition and diversity across zones. (a) Relative abundance of dominant bacterial phyla and archaeal classes in each sample; phylum names follow currently valid nomenclature. (b) PCoA of Bray–Curtis dissimilarities among samples; ellipses are 95% confidence regions. (c) Between- and within-zone Bray–Curtis dissimilarities, with pairwise ANOSIM *R* (***P < 0.001, Bonferroni-corrected) and global PERMANOVA; homogeneity of multivariate dispersion among zones was assessed with betadisper (*F* = 58.52, *P* = 0.0001). (d) Shared and unique ASVs among zones. (e, f) Observed ASV richness and Shannon diversity. (g, h) Within-zone unweighted UniFrac and Jaccard dissimilarities. In panels (e, f), Kruskal–Wallis tests were followed by two-sided Dunn’s post hoc tests with Bonferroni correction (*P < 0.05, **P < 0.01, ***P < 0.001, ****P < 0.0001; ns, not significant). In panels (g, h), differences in multivariate dispersion among zones were tested with betadisper on distances to group centroids (*F* and *P* shown; letters denote groups that differ at *P* < 0.05 after Bonferroni correction); the corresponding test for Bray–Curtis is given in (c). Boxes show medians and interquartile ranges; whiskers extend to 1.5 × IQR.

Community composition differed among the four zones (Fig. 3b). Pairwise ANOSIM indicated the weakest differentiation between the two fresh groundwater transects and stronger differentiation in all comparisons involving the transition zone or lake water (E vs. W, *R* = 0.375; W vs. M, *R* = 0.907; E vs. M, *R* = 0.999; E vs. L, *R* = 1.000; W vs. L, *R* = 0.998; M vs. L, *R* = 0.999; all *P* = 0.0001), and PERMANOVA attributed 45.7% of the variation in community composition to zone membership (*F* = 23.59, *P* = 0.0001). Multivariate dispersion also differed among zones (betadisper, *F* = 58.52, *P* = 0.0001): lake water had the lowest mean distance to the group centroid (0.279, compared with 0.443–0.566 in the other zones), all pairwise dispersion comparisons involving lake water were significant after Bonferroni correction, and dispersion did not differ significantly between the two fresh groundwater transects. Dispersion also differed among zones for unweighted UniFrac (*F* = 11.12, *P* = 0.0001) and Jaccard distances (*F* = 27.70, *P* = 0.0001); for both metrics the two fresh groundwater transects did not differ, whereas the transition zone and lake water were less dispersed than either transect (Bonferroni-corrected pairwise tests). These differences in dispersion require caution when interpreting the ANOSIM and PERMANOVA results as evidence of differences in group location alone.

The distribution of individual ASVs further highlighted compositional differences among zones. Only 108 ASVs (0.49%) were detected in all four zones, whereas 10 448 were detected exclusively in fresh western groundwater, 5102 in fresh eastern groundwater, 2072 in lake water, and 1365 in the transition zone (Fig. 3d). The largest set shared exclusively by two zones was that of the two fresh groundwater transects (1381 ASVs), followed by western groundwater and the transition zone (525 ASVs); fewer than 300 ASVs were shared exclusively between lake water and any groundwater zone.

Alpha diversity differed among zones, with lake water showing the lowest richness and Shannon diversity. Median observed ASV richness was highest in fresh western groundwater (1247), followed by the transition zone (818), fresh eastern groundwater (817), and lake water (183); median Shannon diversity followed the same order (4.53, 3.99, 3.84, and 3.21). Differences among zones were significant for both measures (Kruskal–Wallis *P* = 1.1 × 10⁻^14^ and 4.3 × 10⁻^9^, respectively; Fig. 3e and f). Within-zone heterogeneity also varied: fresh western groundwater had the highest median values for all three metrics (Bray–Curtis 0.83, unweighted UniFrac 0.68, Jaccard 0.91), whereas the lowest values occurred in lake water for Bray–Curtis (0.38) and Jaccard (0.67) and in the transition zone for unweighted UniFrac (0.53) (Fig. 3c, g, h).

### Zone-associated indicator taxa

Indicator analysis identified 392 ASVs significantly associated with individual zones (*r*_g_ > 0.3; Benjamini–Hochberg-adjusted *P* < 0.05). Their distribution was highly uneven: 350 were associated with the transition zone, compared with 33 for fresh eastern groundwater, 6 for fresh western groundwater, and 3 for lake water (Fig. 4a); all indicator taxa are listed in Supplementary Data 6. Although associations with both individual zones and combinations of zones were evaluated, all significant associations were with single zones; none involved a combination of zones.

**Figure 4:**
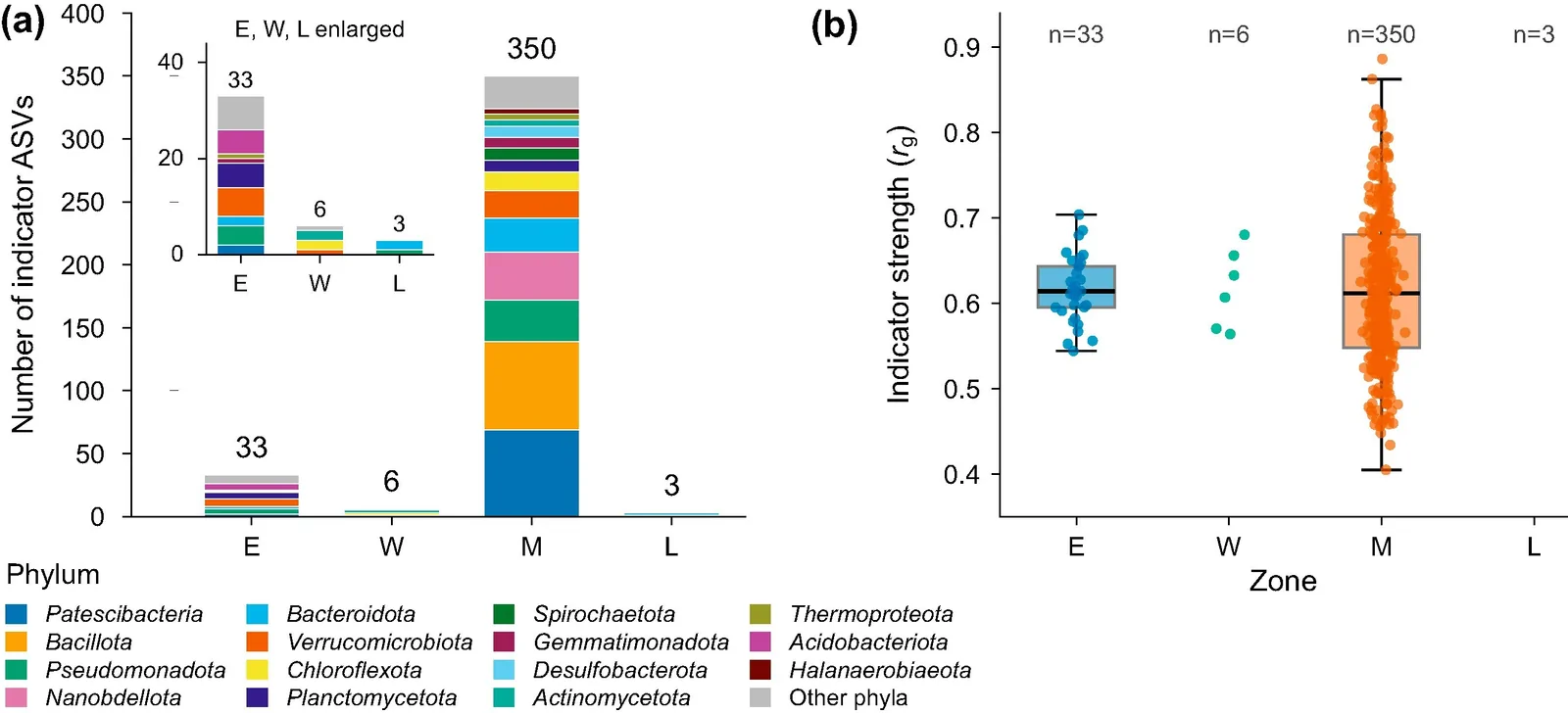
Zone-associated indicator taxa. (a) Number of indicator ASVs identified for each zone (*r*_g_ > 0.3, Benjamini–Hochberg-adjusted *P* < 0.05), colored by phylum; the inset enlarges E, W, and L. (b) Indicator strength (*r*_g_) of the ASVs assigned to each zone; boxes show medians and interquartile ranges. The identity of every indicator ASV is listed in Supplementary Data 6.

The taxonomic composition of these indicator sets differed among zones. Transition-zone indicators were drawn from 31 phyla, led by *Bacillota* (70 ASVs, 20.0%), *Patescibacteria* (69, 19.7%), and *Nanobdellota* (38, 10.9%). Eastern groundwater indicators were dispersed across 14 phyla, with *Verrucomicrobiota* (6 ASVs), *Acidobacteriota* (5), and *Planctomycetota* (5) contributing most; they included ASVs assigned to Nitrosarchaeum and Nitrospira, as well as three Lacunisphaera ASVs (Supplementary Data 6). The six western groundwater indicators comprised two *Chloroflexota* (class *Dehalococcoidia*), two *Actinomycetota*, one *Verrucomicrobiota* (order *Omnitrophales*), and one *Iainarchaeota*. The three lake-water indicators comprised two *Psychroflexus* ASVs (*Bacteroidota*) and one *Halomonadaceae* ASV (*Pseudomonadota*).

Median *r*_g_ values were similar across zones (transition zone, 0.612; fresh eastern groundwater, 0.614; fresh western groundwater, 0.620; and lake water, 0.681), indicating that zones differed in the number of indicator taxa rather than in how strongly individual taxa were associated with them (Fig. 4b). The transition zone contained 58 taxa with *r*_g_ of at least 0.7, whereas no other zone contained more than one. We therefore make no interpretive distinction between zones on the basis of indicator strength.

### Microbial community assembly processes and environmental associations

Mantel correlograms indicated significant phylogenetic signal at short evolutionary distances (Fig. S3), supporting the use of βNTI-based null models. Assembly processes shifted along the continuum (Fig. 5a). Across all 88 samples, dispersal limitation was by far the most prevalent process (59.6% of 3828 pairwise comparisons), followed by variable selection (17.2%), homogeneous selection (12.3%), undominated processes (7.3%), and homogenizing dispersal (3.7%).

**Figure 5:**
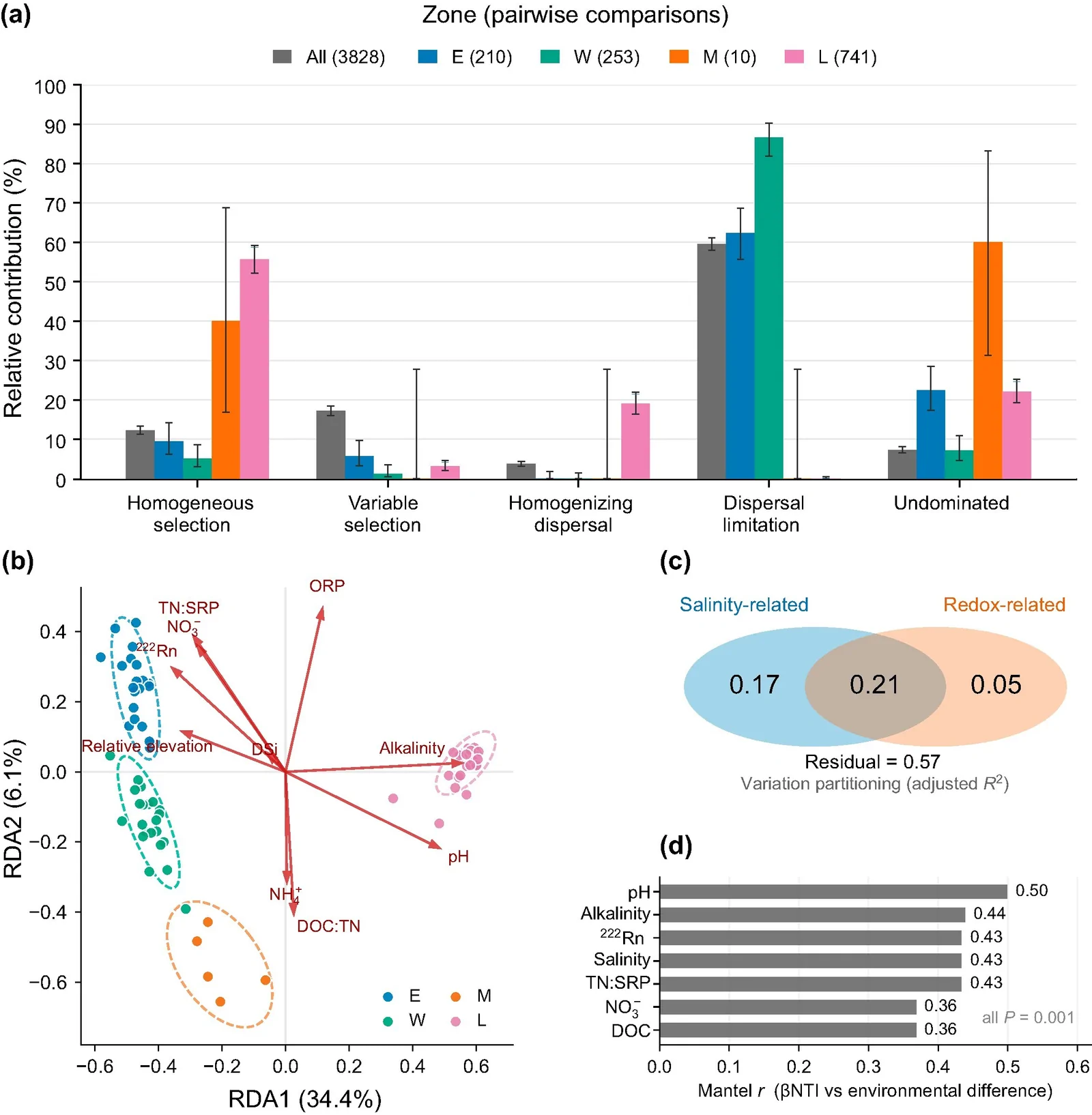
Community assembly processes and environmental correlates. (a) Relative contribution of assembly processes to pairwise comparisons across the whole system and within each zone [βNTI and abundance-based Raup–Crick (RCbray) null models; number of comparisons in parentheses; error bars, approximate Wilson 95% confidence intervals (see Methods)]. Absolute values depend on the ASV filtering criterion, but the contribution of selection remained higher in the transition zone and lake water than in either groundwater transect under all criteria (Supplementary Data 7). (b) RDA of Hellinger-transformed ASV abundances constrained by the environmental variables retained by forward selection; axes show the proportion of total variance explained. (c) Variation partitioning between salinity-related and redox-related variables (adjusted *R*²). (d) Mantel correlations between pairwise βNTI and environmental differences. Abbreviations as in Fig. 2.

Within zones, dispersal limitation remained dominant in the groundwater transects but was absent in the transition zone and lake. Both groundwater transects were dominated by dispersal limitation (eastern, 62.4%; western, 86.6%), with undominated processes secondary (eastern, 22.4%; western, 7.1%) and homogenizing dispersal essentially absent. The transition zone was split between undominated processes (60.0%) and homogeneous selection (40.0%), with no comparison assigned to dispersal limitation, although with only 10 pairwise comparisons this partitioning is necessarily imprecise. Lake-water communities were structured mainly by homogeneous selection (55.7%), together with undominated processes (22.1%) and homogenizing dispersal (19.0%), with a smaller contribution from variable selection (3.1%) and, again, no dispersal limitation. Because the within-lake βNTI distribution is centered close to the −2 threshold (median −2.10), the share assigned to homogeneous selection is sensitive to small changes in the data, although the absence of dispersal limitation held under all filtering criteria tested.

Deterministic selection, comprising homogeneous and variable selection, increased along the continuum, accounting for 15.2% of comparisons in fresh eastern groundwater, 6.3% in fresh western groundwater, 40.0% in the transition zone, and 58.8% in lake water. In contrast to this selection gradient, dispersal limitation was concentrated in the low-salinity groundwater and was absent from the transition zone and lake. The estimated contributions were sensitive to the ASV filtering criterion applied before null modeling: replacing the mean relative abundance threshold of 1 × 10⁻^4^ (670 ASVs) with an occurrence threshold of at least five samples (2 857 ASVs) raised the estimated contribution of selection in every zone. In western groundwater selection rose from 6.3% to 55.7%, and in the lake from 58.8% to 90.9%. The transition zone and lake water nonetheless showed larger deterministic shares than either groundwater transect under all three criteria (Supplementary Data 7).

Environmental-driver analyses were conducted across the four zones using the 84 samples with complete environmental records. In one-variable analyses, alkalinity alone accounted for an adjusted *R*² of 0.333. Forward selection retained 10 environmental variables (adjusted *R*² = 0.470, compared with 0.477 for the full set of 17; Fig. 5b). Salinity and alkalinity were almost perfectly collinear (*r* = 0.999). Although each explained the largest share when fitted alone, marginal tests in the full 17-variable model attributed no statistically significant unique contribution to either. Variation partitioning with these variables treated as groups assigned a unique adjusted *R*² of 0.17 to salinity-related and 0.05 to redox-related variables, with 0.21 shared and 0.57 unexplained (Fig. 5c). Pairwise βNTI correlated most strongly with differences in pH (Mantel *r* = 0.50), followed by TN:SRP, ^222^Rn, alkalinity and salinity (*r* = 0.43–0.44; all *P* = 0.001; Fig. 5d), although these associations were modest overall. Geographic distance added little beyond environment: community dissimilarity increased with distance across the system (Mantel *r* = 0.26, *P* = 0.0001; Fig. S4a), but the relationship weakened substantially, although it remained significant, after controlling for environmental dissimilarity (partial Mantel *r* = 0.06, *P* = 0.020); within the subsurface a weak partial relationship remained (*r* = 0.14, *P* = 0.004; Fig. S4b).

### Predicted functional profiles

Predicted functional profiles distinguished the four zones (PCo1 = 51.8%, PCo2 = 11.5%, based on KO relative abundances; Fig. 6a), with the first axis separating lake water from all groundwater samples. Predicted KO abundances are provided in Supplementary Data 8 and predicted MetaCyc pathway abundances in Supplementary Data 9.

**Figure 6:**
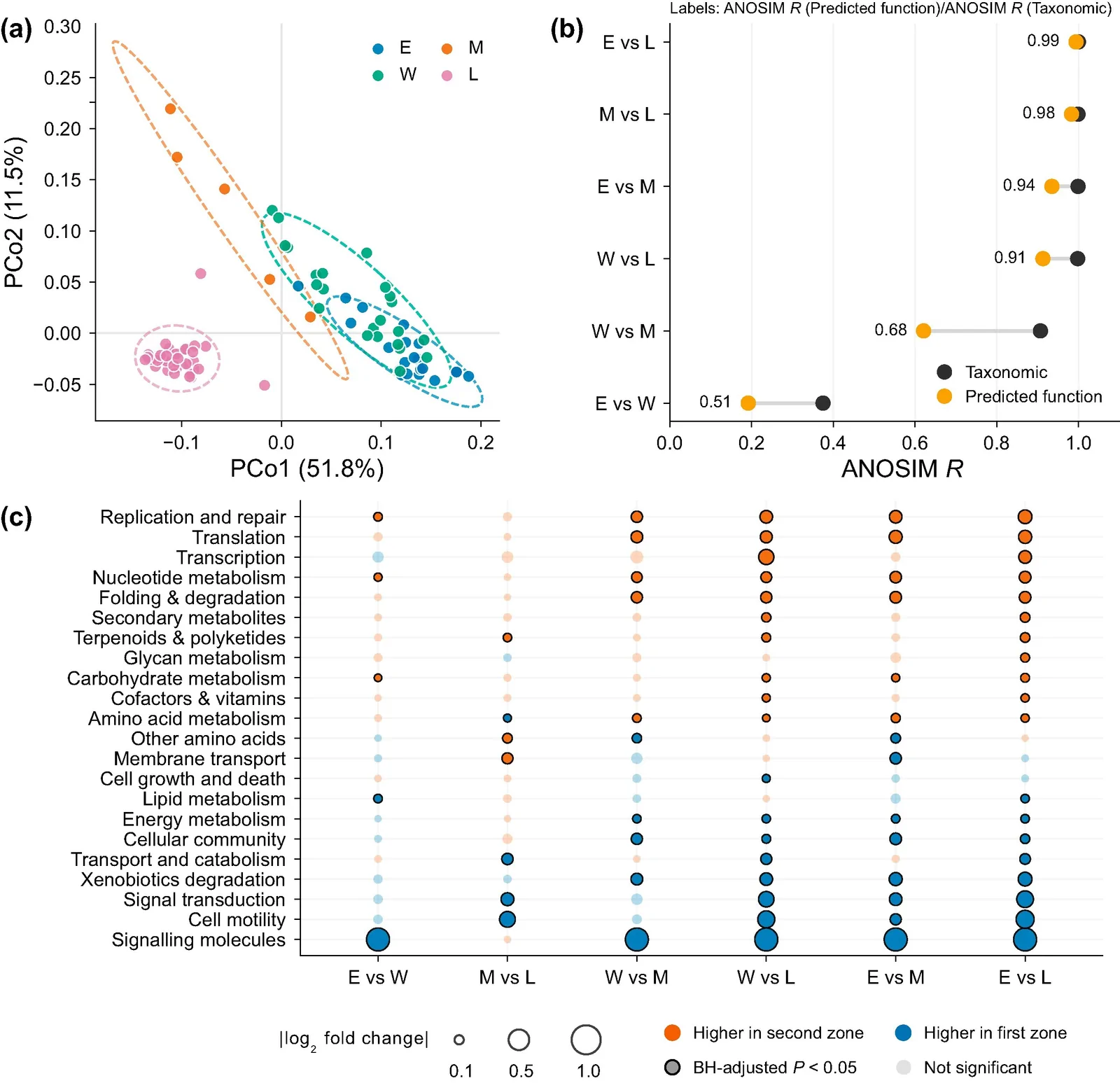
Predicted functional profiles inferred by PICRUSt2. (a) PCoA (Bray–Curtis) of predicted KO (KEGG orthologue) relative abundances. (b) Pairwise ANOSIM *R* for taxonomic and predicted functional profiles; numbers give the ratio of predicted-functional to taxonomic ANOSIM *R*. (c) Between-zone differences in the predicted abundance of KEGG Level 2 functional categories, indicating for each pairwise comparison whether a category is more abundant in the first or second zone listed (see key). Circle size scales with |log₂ fold change|; outlined circles, Benjamini–Hochberg-adjusted *P* < 0.05. Functional profiles are 16S rRNA gene-based predictions of potential, not realized, capacity.

Functional profiles differed significantly between every pair of zones, including the two fresh groundwater transects (ANOSIM *R* = 0.192, *P* = 0.0003). Differentiation was nonetheless consistently weaker than in taxonomic composition: the ratio of functional to taxonomic ANOSIM *R* was 0.51 between the two groundwater transects, 0.68 between western groundwater and the transition zone, and 0.91–0.99 for the remaining pairwise comparisons (Fig. 6b). The contrast between functional and taxonomic differentiation was therefore most pronounced between the two fresh groundwater transects.

At KEGG Level 2, 19 of 22 functional categories differed significantly between fresh eastern groundwater and lake water and 18 between fresh western groundwater and lake water, compared with only five between the two groundwater transects (Fig. 6c). Relative to lake water, both groundwater transects were enriched in predicted pathways associated with cell motility, xenobiotics biodegradation, signal transduction, and transport and catabolism, whereas lake water was enriched in pathways associated with replication and repair, translation, and nucleotide metabolism. Effect sizes between the two groundwater transects were generally smaller than those for groundwater–lake comparisons (median |log_2_ fold change| among significant categories 0.07 vs. 0.18); full results are given in Supplementary Data 10.

## Discussion

### Hydrogeochemical structure of the continuum

The groundwater–hypersaline lake continuum exhibits pronounced hydrogeochemical zonation controlled by evaporative concentration, groundwater flow-path characteristics, and sediment–water interactions (Zhang et al. 2021a). This multi-dimensional architecture provides the physicochemical context for microbial habitat differentiation across the continuum (Fig. 2a).

Despite sharing similarly low salinities, the two groundwater transects diverge in redox conditions. The reducing conditions in western groundwater (negative ORP, nitrate depletion, and elevated SRP and DSi) are consistent with the influence of organic-rich paleolake sediments along the flow path (Zhang et al. 2021a). Organic matter in these fine-grained deposits could promote oxygen consumption and anaerobic respiration, in contrast to the more oxidizing eastern aquifer (McMahon and Chapelle 2008, Kolbe et al. 2019). This contrast suggests that sediment composition and depositional history contribute to redox differentiation within the groundwater domain, although sediment properties and microbial reaction rates were not measured directly in this study.

The transition zone shows non-conservative chemistry consistent with in situ biogeochemical transformation rather than conservative mixing: NH₄⁺ and SRP concentrations exceeded those in both adjacent zones, and together with strongly reducing conditions they are consistent with intensive organic matter remineralization. Reductive dissolution of iron- and manganese-bearing phases could mobilize sediment-bound phosphorus, while dissimilatory processes could contribute to ammonium accumulation (Triska et al. 1993, Boano et al. 2014). Sharp minima in TN:SRP and DSi:SRP ratios are consistent with preferential phosphorus mobilization under reducing conditions, highlighting the hyporheic-like character of this groundwater–surface water interface.

Despite receiving groundwater inputs, lake stoichiometry is reshaped by internal cycling and by atmospheric exchange through evaporation and degassing. The resulting chemistry differs markedly from that of the source water, with hyper-accumulation of alkalinity and DOC alongside divergent nutrient stoichiometry. Such divergence between source water and lake chemistry is characteristic of groundwater-fed terminal lakes in arid regions (Wurtsbaugh et al. 2017).

The two principal components are consistent with these two levels of control (Fig. 2): a coupled salinity–alkalinity–organic matter axis associated with evaporative concentration toward the terminal lake (Soetaert et al. 2007, Getenet et al. 2023, Zhang et al. 2024), and a redox-related axis separating the more oxidizing eastern groundwater from the more reducing western groundwater and transition zone, consistent with heterogeneity in sedimentary properties and organic matter supply (Triska et al. 1993, Boano et al. 2014, Kuypers et al. 2018). Although the PCA axes are orthogonal by construction, this does not imply that the underlying environmental processes operate independently. Variation partitioning of community composition identified a shared fraction between the salinity- and redox-related predictor groups that exceeded either unique fraction, indicating that the community variation associated with each group overlaps substantially (Section “Microbial community assembly processes and environmental associations”).

Together, these results indicate that hydrogeochemical organization is governed by hierarchical controls. Salinity and alkalinity act as system-scale drivers associated with evaporative concentration along a hydrologically connected flow path, while sedimentary legacy and organic matter availability impose secondary redox constraints within specific groundwater flow paths. This multiscale structure provides the physicochemical template upon which microbial communities respond through differential selection, dispersal, and predicted functional potential (Zhou et al. 2014).

### Community differentiation along the continuum

Microbial community differentiation closely tracked hydrogeochemical zonation: communities were strongly differentiated among zones and lake water supported the lowest diversity (Fig. 3). The taxa driving this differentiation reflect two distinct constraints: redox conditions within the subsurface and osmotic stress in the lake. Fresh western groundwater and the transition zone are enriched in *Bathyarchaeia* and *Desulfobacterota* relative to fresh eastern groundwater and lake water (Fig. S2). These lineages are characteristically anaerobic and are associated with fermentation and sulfate reduction, processes that promote reductive dissolution of Fe/Mn oxides and associated phosphorus release (Boano et al. 2014, Kuypers et al. 2018). Within the groundwater domain, the two flow paths differed in a manner consistent with their contrasting sedimentary settings, with the more oxidizing eastern groundwater favoring predatory *Bdellovibrionota* (Liau et al. 2022). At the hypersaline end, communities narrowed to salt-adapted and stress-tolerant lineages (*Halobacteria, Actinomycetota*, and *Bacillota*), while groundwater-associated taxa (*Nanoarchaeia, Patescibacteria*) were depleted, consistent with strong environmental filtering under extreme osmotic stress (Oren 2011). Although fresh western groundwater and the transition zone are strongly differentiated in overall composition (ANOSIM *R* = 0.907; Fig. 3b), their shared enrichment in anaerobic lineages reflects a common redox setting—a taxon-level signature of reducing subsurface conditions.

The magnitude of differentiation was highly uneven. The two fresh groundwater transects were the least distinct pair in the system (ANOSIM *R* = 0.375), whereas every comparison involving the transition zone or the lake yielded *R* ≥ 0.907. Compositional separation therefore tracks the salinity gradient rather than geographic proximity: the eastern and western transects lie on opposite sides of the lake yet resemble each other more closely than either resembles the transition zone immediately adjacent to the western wells. Consistent with this, only 0.49% of ASVs were shared across all four zones (Fig. 3d), and lake water was almost completely separated from every other zone (*R* ≥ 0.998). Despite continuous hydrological flow, the energetic cost of osmoregulation provides a plausible explanation for the failure of groundwater taxa to establish in the lake (Oren 2011), so that hydrological connectivity does not translate into biological connectivity. The few ASVs detected in all four zones fit this pattern. Most of the 108 shared ASVs were represented outside their main zone only at trace level: the salt-adapted lineages that dominate the lake (*Halomonas, Spiribacter*, and *Psychroflexus*) were detected in fresh groundwater at only 0.3%–2.2% of their total reads, and conversely the *Pseudomonas* ASVs that dominate groundwater had only 0.7% of their reads in lake water (Supplementary Data 3). These detections may represent transported cells, extracellular DNA, or rare resident populations; in each case, the lineage was only marginally represented outside the zone it dominates, consistent with strong local selection at zone boundaries.

Diversity and within-zone heterogeneity also differed among zones. Reducing western groundwater supported the highest alpha diversity and the greatest within-zone dissimilarity for all three metrics (Fig. 3c, e–h), which may reflect finer-scale environmental heterogeneity within that transect, although sediment properties were not measured directly and this link cannot be established from our data. Lake communities had the lowest richness, the lowest multivariate dispersion (mean distance to centroid 0.279), and the lowest within-zone Bray–Curtis and Jaccard dissimilarities, although the lowest unweighted UniFrac distance occurred in the transition zone. This combination is consistent with strong environmental filtering under uniformly hypersaline conditions.

### The transition zone as a selective environment

The transition zone did not simply contain a mixture of taxa from the adjacent groundwater and lake, but supported a distinct assemblage of its own. Of the 392 indicator ASVs identified across the system, 350 were associated with the transition zone, compared with 33, 6, and 3 for fresh eastern groundwater, fresh western groundwater and lake water, respectively (Fig. 4a). A passive mixture would be expected to yield few taxa distinctively associated with the zone, since its communities would be drawn from those on either side; instead, the transition zone supported by far the largest set of zone-specific indicator taxa in the system.

The identity of these taxa is consistent with the conditions found there. Transition-zone indicators were drawn from 31 phyla and led by *Bacillota* (70 ASVs, 20.0%), *Patescibacteria* (69, 19.7%), and *Nanobdellota* (38, 10.9%). *Bacillota* are noted for tolerance of fluctuating salinity (Hou et al. 2021, Wang et al. 2025), while the genome-reduced *Patescibacteria* and *Nanobdellota* are thought to depend on fermentation or on obligate associations with other cells (Wurch et al. 2016, Jarett et al. 2018, Herrmann et al. 2019, Tian et al. 2020). This combination of stress tolerance and metabolic dependency is characteristic of a habitat that is chemically severe but organically rich, matching the strongly reducing, ammonium- and phosphorus-enriched conditions measured in this zone.

The contrast with the adjacent groundwater is informative. Fresh western groundwater supported the highest ASV richness in the system, yet yielded only six indicator taxa after correction for multiple testing: two *Chloroflexota* (*Dehalococcoidia*), two *Actinomycetota*, one *Verrucomicrobiota* (*Omnitrophales*), and one *Iainarchaeota*, several of which are associated with anoxic subsurface conditions. A species-rich community need not yield many indicator taxa if no single taxon is consistently associated with the zone; the high within-zone heterogeneity of this transect (Fig. 3c) would also reduce the consistency required for detection. The three lake-water indicators, comprising two *Psychroflexus* ASVs and one *Halomonadaceae* ASV, represent the opposite case: a narrow, physiologically specialized assemblage in which few taxa suffice to characterize the habitat.

These differences reflect the number of indicator taxa rather than the strength of their association. Median *r*_g_ values were similar across the four zones (0.612–0.681), and we therefore make no interpretive distinction between zones on this basis. The transition zone also contained 58 taxa, 16.6% of its indicator set, with *r*_g_ of at least 0.7, whereas no other zone contained more than one. Within the zone, homogeneous selection accounted for four of the 10 pairwise comparisons and undominated processes for the remaining six (Section “Microbial community assembly processes and environmental associations”), so local selection operates alongside outcomes that no single process dominates, consistent with a small, chemically dynamic interface. Taken with its distinctive chemistry and its large indicator set, this is consistent with the transition zone acting as a distinct selective environment at the subsurface–surface boundary rather than one shaped by mixing alone.

### From dispersal limitation to selection across the groundwater–hypersaline lake continuum

Dispersal limitation was the most prevalent process across the system as a whole (59.6% of pairwise comparisons) and remained dominant within the two low-salinity groundwater transects (eastern, 62.4%; western, 86.6%). It was not, however, a system-wide constant: dispersal limitation fell to zero in the transition zone and the lake, where selection and undominated processes prevailed. This pattern is not explained by geographic distance alone: after controlling for environmental dissimilarity, community composition was only very weakly related to distance across the system (partial Mantel *r* = 0.06; Fig. S4).

Within the subsurface, communities were structured by dispersal limitation, consistent with limited advective mixing over the tens of meters separating wells, although groundwater velocities were not measured; under such conditions, restricted dispersal and drift can generate compositional divergence among samples (Griebler and Lueders 2009). Contrary to the common expectation that hydrological connectivity homogenizes aquifer communities through mass effects (Leibold et al. 2004, Griebler and Lueders 2009), connectivity here did not translate into microbial mixing even within a single transect (Danczak et al. 2018). Dispersal limitation was even stronger in the western transect (86.6%) than in the eastern (62.4%), consistent with the finer-grained, silt-rich western sediments impeding transport more than the permeable eastern sands; the same west–east sedimentary contrast that underlies the redox differentiation (Section “Hydrogeochemical structure of the continuum”) therefore also grades the strength of dispersal limitation, so sedimentary legacy may act simultaneously as a redox filter and a dispersal barrier. Undominated processes were correspondingly more prevalent in the eastern transect (22.4% vs. 7.1%). Undominated outcomes arise where no single process leaves a consistent signature, which may occur where opposing processes act simultaneously or where their effects vary among sample pairs.

Superimposed on this pattern, the balance of assembly shifted toward selection along the salinity gradient. Deterministic selection (combining homogeneous and variable selection) accounted for a progressively larger share of assembly toward the hypersaline end of the continuum, from 6.3% in fresh western groundwater and 15.2% in fresh eastern groundwater to 40.0% in the transition zone and 58.8% in lake water (Fig. 5a). This selection was almost entirely homogeneous rather than variable (variable selection ≤6% within any zone), indicating a spatially uniform filter within each zone rather than divergent selection among sites. Under every ASV filtering criterion tested, the transition zone and lake water showed larger deterministic shares than either groundwater transect, although the absolute values varied (Supplementary Data 7), and we therefore base our interpretation on this contrast alone. The pattern is consistent with our first hypothesis: as ionic stress intensifies, the range of viable physiologies narrows and selection leaves an increasingly consistent signature. This represents a stochastic-to-deterministic transition along a single connected gradient, consistent with the general expectation that environmental harshness strengthens deterministic assembly, with the hypersaline endpoint imposing the narrowest physiological filter (Chase 2010, Dini-Andreote et al. 2015).

At the hypersaline endpoint, strong selection was not accompanied by restricted dispersal: homogeneous selection accounted for 55.7% of within-lake comparisons, undominated processes for 22.1%, homogenizing dispersal for 19.0% and variable selection for 3.1%, with no comparison assigned to dispersal limitation. This contrasts with other extreme environments, where dispersal limitation is often substantial (Song et al. 2022, Lemoine et al. 2023, Liu et al. 2025). The contrast is particularly pronounced for hadal sediments, where homogeneous selection and dispersal limitation contribute almost equally (50.5% and 43.8%) (Xiao et al. 2025), although differences in ASV filtering and null-model implementation limit direct comparison of percentages among studies. Within our lake, the shallow water column offers a straightforward explanation: continual mixing sustains dispersal and erodes compositional differences among sites (within-zone Bray–Curtis dissimilarity 0.38, the lowest in the system). More broadly, assembly process and within-zone heterogeneity co-vary across the continuum: the dispersal-limited groundwater transects show the greatest compositional divergence (western Bray–Curtis dissimilarity 0.83) and the selection-dominated lake the least, so restricted dispersal generates divergence in the subsurface whereas uniform selection homogenizes the surface.

At the scale of the whole continuum, the process architecture we observe is not unique to this system: dispersal limitation dominates the low-salinity subsurface, and selection intensifies toward the hypersaline endpoint. A related pattern appears in estuarine salinity gradients, the classic setting for microbial studies along salinity transitions. In estuarine and shelf sediments of the northern South China Sea, assembly is likewise dominated by stochastic processes, and selection strengthens toward the more environmentally uniform shelf (Lu et al. 2022). The stochastic component, however, differs with the hydrodynamic regime: in estuarine water columns, tidal mixing continually redistributes taxa, favoring homogenizing dispersal (Crump et al. 2004, Fortunato et al. 2012), whereas in our system the slow, unidirectional groundwater flow instead limits dispersal within the subsurface, where the gradient is compressed into tens of meters.

Environmental variables accounted for only part of this structure. Across the system, community composition was associated primarily with the salinity–alkalinity complex: forward selection retained 10 variables, alkalinity fitted as a single predictor accounted for an adjusted *R²* of 0.333, and salinity-related variables accounted for a unique adjusted *R*² more than three times that of redox-related variables (Fig. 5c). Pairwise βNTI correlated most strongly with differences in pH, TN:SRP, ^222^Rn, alkalinity, and salinity, but these correlations were modest, indicating that the measured geochemistry only partly tracks which process dominates in a given comparison.

### Predicted functional convergence tracks environmental similarity

Predicted functional profiles broadly tracked taxonomic patterns, but differentiation among zones was consistently weaker than in taxonomic composition. Principal coordinate analysis resolved a primary separation between the hypersaline lake and all groundwater communities, with more limited differentiation between the eastern and western flow paths (Fig. 6a). All functional inferences below derive from 16S rRNA gene-based prediction and describe potential rather than realized metabolic capacity.

The contrast between functional and taxonomic differentiation varied among zone pairs. The ratio of functional to taxonomic ANOSIM *R* was 0.51 between the two fresh groundwater transects, 0.68 between western groundwater and the transition zone, and 0.91–0.99 for the remaining pairwise comparisons (Fig. 6b). The contrast was therefore greatest between the two fresh groundwater transects and smallest once the salinity gradient was crossed. This pattern is consistent with taxonomically distinct communities retaining similar predicted repertoires under shared environmental conditions, whereas stronger environmental contrasts coincide with differentiation in both composition and predicted function (Fierer et al. 2012, Louca et al. 2016).

The two groundwater transects exemplify this. They differ substantially in taxonomic composition and in redox conditions, yet their predicted gene content is only weakly differentiated: the few significant differences at KEGG Level 2 were small (median |log_2_ fold change| 0.07), apart from one large difference in a sparsely populated category (signaling molecules and interaction). We therefore describe the two transects as functionally similar rather than functionally identical, and note that this similarity does not establish functional redundancy. Predicted gene content reflects the metabolic potential encoded in a community, not which of those capacities are expressed (Douglas et al. 2020); two assemblages with similar predicted repertoires may still differ in their realized activity (Martiny et al. 2013). That the two transects converge functionally despite contrasting redox conditions suggests that redox adaptation resides largely in regulation or in accessory functions not captured by 16S rRNA gene-based prediction, rather than in the core gene repertoire—a possibility that metagenomics could test.

Across the salinity gradient, in contrast, the predicted differences were both numerous and large. Groundwater communities were enriched in pathways for cell motility, xenobiotics biodegradation, signal transduction, and transport and catabolism, consistent with adaptation to oligotrophic subsurface conditions in which resources must be located and acquired (Griebler and Lueders 2009). The enrichment of predicted xenobiotic degradation pathways is compatible with utilization of recalcitrant organic carbon derived from paleolake sediments, although direct evidence would require metagenomic or process-rate measurements. Lake communities were instead enriched in replication and repair, translation, and nucleotide metabolism (Bodaker et al. 2010, Ionescu et al. 2012), differences that indicate a shift in the relative representation of predicted cellular functions under hypersaline conditions but do not by themselves establish higher rates of growth or repair.

Taken together, these predicted profiles are consistent with a reorganization of metabolic strategy along the continuum, from broad repertoires suited to locating scarce resources in the subsurface to a narrower emphasis on maintenance and replication-related functions under sustained osmotic stress. That functional differentiation was weaker than taxonomic differentiation within the subsurface further suggests that compositionally distinct groundwater communities converge on similar predicted repertoires, whereas crossing into hypersaline conditions requires a genuinely different functional complement.

### An integrative framework for microbial organization

Integration of hydrogeochemical gradients, community composition, indicator taxa, assembly processes, and predicted functional capacity establishes a process-based framework for microbial organization along the groundwater–hypersaline lake continuum (Fig. 7). Several features distinguish this system from a simple linear response to salinity. Assembly shifts along the continuum, from dispersal limitation dominating the low-salinity subsurface to selection dominating the transition zone and hypersaline lake. The transition zone is not simply an intermediate mixture but a zone of distinctive local selection. Predicted functional differentiation was weaker than taxonomic differentiation, most markedly within the subsurface. As a result, taxonomic and functional differentiation partly decouple there, but not across the salinity gradient. Finally, the taxa detected across all zones are shared almost exclusively at trace level, without establishing more than a marginal presence outside the zones they dominate.

**Figure 7:**
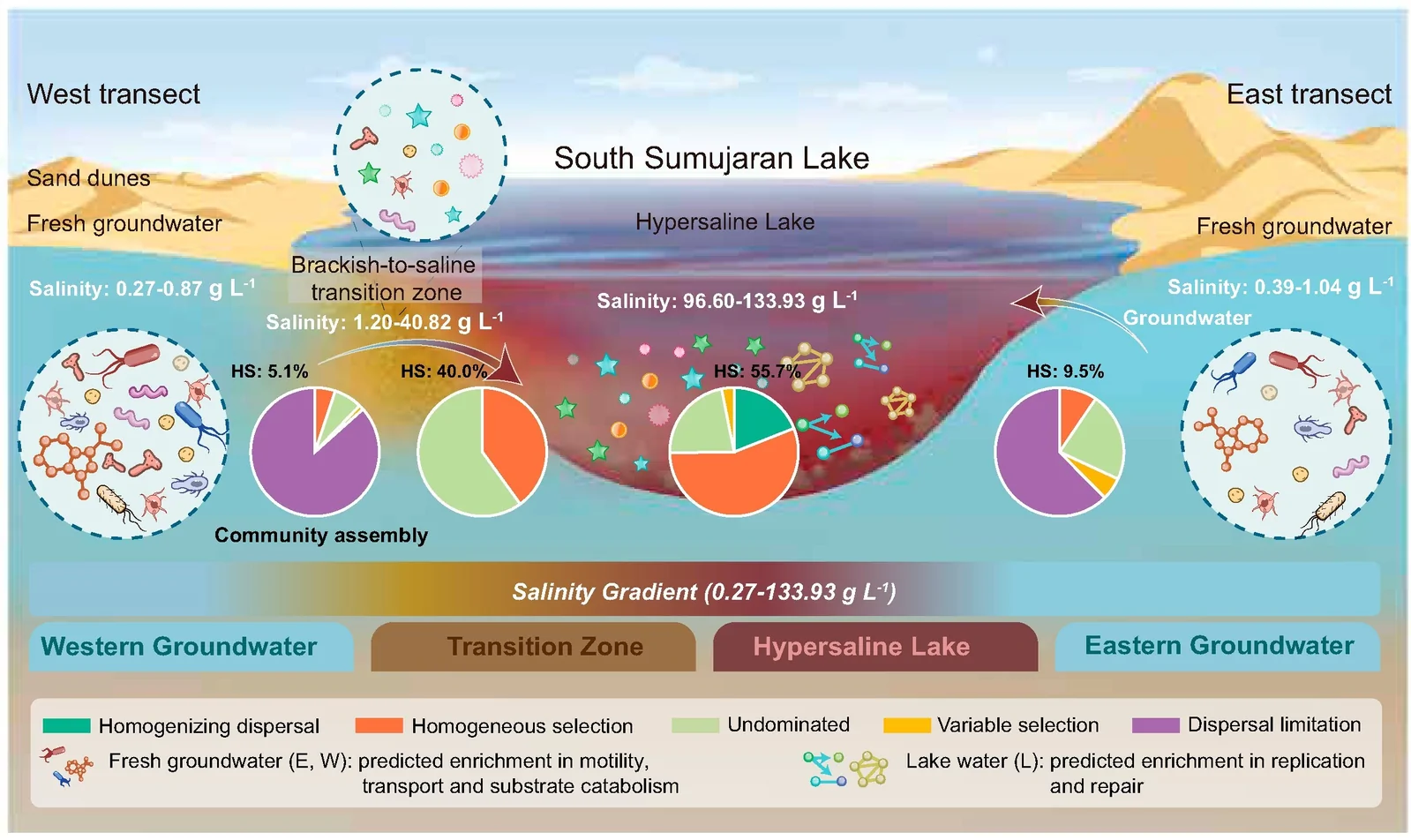
Conceptual summary of community assembly across the groundwater–hypersaline lake continuum. Groundwater flows from two transects toward the terminal lake across a more than 300-fold salinity gradient. Pie charts show the contribution of each assembly process within each zone, with the share of homogeneous selection labeled. Functional labels summarize PICRUSt2-predicted enrichment (Fig. 6) and do not represent measured activity.

Together these features delineate a hierarchical model in which salinity imposes first-order constraints at the system scale, sedimentary legacy is associated with secondary differences in assembly within the groundwater domain, and the subsurface–surface boundary constitutes a distinct selective environment. More generally, the physical template that concentrates solutes also partitions the two fundamental assembly forces in space: dispersal limitation governs the slow-flow subsurface, whereas selection governs the geochemically extreme surface, so that a single hydrological continuum encompasses a spatial transition from stochastic to deterministic control.

### Limitations and future directions

This study represents a spatial snapshot of a single continuum, and generalization to other groundwater-fed terminal lakes will require comparable datasets from independent basins. Seasonal and interannual variability may alter redox structure and mixing intensity, so time-resolved observations will be needed to assess the stability of the organization inferred here.

A transition zone was sampled adjacent to only one of the two groundwater transects, so the relative connectivity of the eastern and western systems cannot be evaluated from our design. Within the subsurface, community dissimilarity also remained weakly but significantly related to geographic distance after controlling for environmental dissimilarity (partial Mantel *r* = 0.14, *P* = 0.004; Fig. S4b), indicating that spatial proximity is partly confounded with zone identity. We therefore confine our interpretation of the transition zone to its own assembly and indicator characteristics. In addition, samples collected at different depths within the same well or lake station are not fully independent, and permutation tests assuming exchangeability may therefore be somewhat anti-conservative. As a check, we repeated the PERMANOVA with zone labels permuted among the 26 wells and lake stations rather than among individual samples; the zone effect remained significant (*F* = 23.59, *P* = 0.0001; Supplementary Code).

Functional inferences derive from 16S rRNA-based gene-content prediction and require metagenomic or metatranscriptomic validation. Coverage is uneven across lineages and is likely least complete for the transition zone, where *Patescibacteria* and *Nanoarchaeia*, groups poorly represented in reference genomes, are abundant; zone-specific assessment of prediction coverage would help quantify this uncertainty.

The absolute contributions of assembly processes depend on how many ASVs are retained before analysis: replacing the primary abundance threshold (670 ASVs) with an occurrence threshold (2857 ASVs) raised the estimated contribution of selection in every zone. Nonetheless, the transition zone and lake water showed larger deterministic shares than either groundwater transect under all three criteria tested (Supplementary Data 7). We therefore base our conclusions on this contrast rather than on absolute percentages. Estimates for the transition zone warrant particular caution because its five samples yield only 10 pairwise comparisons.

## Conclusion

Microbial community assembly along the groundwater–hypersaline lake continuum of South Sumujaran Lake in the Badain Jaran Desert shifted from dispersal limitation to selection along the flow path. Dispersal limitation was the most prevalent process system-wide and occurred within the two low-salinity groundwater transects but not in the transition zone or lake, where homogeneous selection increasingly dominated. Deterministic selection contributed a larger share of assembly in the transition zone and lake water than in either fresh groundwater transect. The transition-zone samples also contained a distinct indicator assemblage. Given the small number of transition-zone wells, however, these patterns should be regarded as suggestive rather than a formal demonstration that local selection outweighs community mixing. Restricted dispersal within the subsurface and steep solute gradients at zone boundaries together structure these communities while hydrological connectivity is maintained. This zone-specific assembly framework helps explain how steep solute gradients structure microbial communities along hydrologically connected flow paths in groundwater-fed terminal lakes of arid regions.

## Supplementary Material

fiag113_Supplemental_Files

## Data Availability

The raw sequence data reported in this paper have been deposited in the Genome Sequence Archive at the National Genomics Data Center, China National Center for Bioinformation/Beijing Institute of Genomics, Chinese Academy of Sciences (GSA: CRA048363) and are publicly accessible at https://ngdc.cncb.ac.cn/gsa. Hydrochemical data and other data supporting the findings of this study are available in the Supplementary Data. The R scripts, input tables, and outputs used for the null-model and indicator-species analyses are provided as Supplementary Code.
