# Supplementary material for: Microbial community assembly along a groundwater–hypersaline lake continuum in the Badain Jaran Desert
Source: FEMS Microbiol Ecol. 2026 Oct 5;102(11):fiag113. doi: 10.1093/femsec/fiag113 (PMC13695145; doi:10.1093/femsec/fiag113)
Supplement: Supplementary file 1 [file fiag113_supplemental_files.zip › SI.docx]

**Supplementary Information of**

**Microbial community assembly along a groundwater–hypersaline lake continuum in the Badain Jaran Desert**

Meiqing Lu^1,2,3,4^, Xiaolang Zhang^5^, Jiu Jimmy Jiao^1,3,4*^, Jinchao Zuo^3^, Hailong Li^6^, and Meng Li^2^

1. CUG-HKU Joint Centre for Coastal Groundwater and Ecosystem, School of Environmental Studies, China University of Geosciences, Wuhan 430078, China

2. Archaeal Biology Centre, Synthetic Biology Research Center, Shenzhen Key Laboratory of Marine Microbiome Engineering, Key Laboratory of Marine Microbiome Engineering of Guangdong Higher Education Institutes, Institute for Advanced Study, Shenzhen University, Shenzhen 518060, China

3. Department of Earth and Planetary Sciences, The University of Hong Kong, Hong Kong, 999077, China

4. Shenzhen Research Institute, The University of Hong Kong, Shenzhen, China

5. Department of Geosciences, Florida Atlantic University, Boca Raton, FL, USA 33431

6. State Key Laboratory of Soil Pollution Control and Safety, Southern University of Science and Technology, Shenzhen, 518055, China

*Corresponding author: Jiu Jimmy Jiao (email: [jjiao@hku.hk](mailto:jjiao@hku.hk))

**Fig. S1. Rarefaction analysis assessing sequencing depth adequacy.** Rarefaction curves of ASV richness (a) and Shannon diversity (b) indicate sufficient sequencing coverage across all samples. The normalization (rarefaction) threshold was set at 61,007 reads per sample.

**Fig. S2. Statistical comparison of the relative abundance of dominant microbial taxa across hydrogeochemical zones**. Panels display the mean relative abundance of major phyla and classes in fresh eastern groundwater (E), fresh western groundwater (W), the brackish-to-saline transition zone (M), and the hypersaline lake (L). Error bars represent standard deviations. Asterisks indicate significant pairwise differences identified by Dunn's post hoc tests following a significant Kruskal–Wallis test, with two-sided *P*-values adjusted by the Bonferroni method (* *P* < 0.05, ** *P* < 0.01, *** *P* < 0.001).

**Fig. S3. Mantel correlogram testing for phylogenetic signal in ASV environmental optima.** The analysis used the 670 ASVs retained for the null-model analysis. The environmental optimum of each ASV was calculated as the abundance-weighted mean of sample scores on the first principal component of the seventeen standardized environmental variables (84 samples with complete records). Mantel correlations between phylogenetic distance and differences in optima were calculated for phylogenetic-distance classes of width 0.02 (999 permutations); filled symbols denote classes significant after Bonferroni correction (adjusted P < 0.05). Significant positive correlations at short phylogenetic distances support the use of phylogenetic turnover in the βNTI framework.

**Fig. S4. Distance decay of community dissimilarity is largely environmentally mediated.** Pairwise Bray–Curtis dissimilarity against geographic distance (Methods) for (a) all 84 samples with complete environmental records and (b) the subsurface zones only (E, W, M). Blue, pairs from the same zone; orange, pairs from different zones. Mantel and partial Mantel statistics (9,999 permutations) are shown in each panel; partial tests control for environmental dissimilarity.

**Table S1.** Ranges and mean values of major physicochemical variables across fresh eastern groundwater (E), fresh western groundwater (W), the western brackish-to-saline transition zone (M), and the hypersaline lake (L) along the groundwater–hypersaline lake continuum of South Sumujaran Lake.

|  | **Salinity**  **(g L^-1^)** | **Alkalinity**  **(mg L^-1^)** | **T**  **(°C)** | **pH** | **ORP**  **(mV)** | **^222^Rn (dpm L^-1^)** | **DOC**  **(mM)** | **TN**  **(mM)** |
| --- | --- | --- | --- | --- | --- | --- | --- | --- |
| **Fresh Eastern Groundwater (E; n = 21)** | | | | | | | | |
| Range | 0.39-1.04 | 50-235 | 14.04-18.78 | 7.44-8.86 | -23.3-114 | 159.44-845.08 | 0.21-1.00 | 0.19-0.91 |
| Mean | 0.49 | 155 | 15.62 | 8.10 | 62.73 | 345.25 | 0.44 | 0.35 |
| **Fresh Western Groundwater (W; n = 23)** | | | | | | | |  |
| Range | 0.27-0.87 | 135-510 | 7.33-16.87 | 7.09-10.49 | -188.9-  -12.1 | 34.68-160.01 | 0.29-1.87 | 0.01-0.12 |
| Mean | 0.40 | 219.57 | 11.18 | 8.59 | -117.47 | 104.63 | 0.78 | 0.04 |
| **Brackish-to-saline Transition Zone (M; n = 5)** | | | | | | | | |
| Range | 1.20-40.82 | 800-16000 | 8.3-11.09 | 9.91-10.49 | -259.6-  -172.9 | 43.05-83.20 | 2.29-6.29 | 0.01-0.03 |
| Mean | 18.05 | 7440 | 10.14 | 10.32 | -221.88 | 68.52 | 3.55 | 0.02 |
| **Hypersaline Lake (L; n = 39)** | | | | | | | | |
| Range | 96.60-133.93 | 30400-42600 | 2.51-21.56 | 10.16-10.36 | -224.8-74.9 | 0.78-85.44 | 0.36-8.81 | 0.15-0.36 |
| Mean | 126.53 | 40628.21 | 10.57 | 10.27 | 4.75 | 15.50 | 6.82 | 0.23 |
|  | **Ammonium (μM)** | **Nitrite**  **(μM)** | **Nitrate (μM)** | **SRP (μM)** | **DSi (μM)** | **DOC:TN** | **TN:SRP** | **DSi:****SRP** |
| **Fresh Eastern Groundwater (E; n = 21)** | | | | | | | | |
| Range | 0-67.56 | 0-135.8 | 149.43-1162.91 | 1.53-7.44 | 170.57-747.22 | 0.49-4.83 | 52.82-170.40 | 44.02-111.68 |
| Mean | 10.42 | 8.72 | 484.72 | 3.25 | 239.73 | 1.43 | 111.07 | 74.94 |
| **Fresh Western Groundwater (W; n = 23)** | | | | | | | | |
| Range | 0-119.12 | 0-3.73 | 0-47.59 | 1.46-8.48 | 125.58-1069.88 | 5.05-188.43 | 1.17-40.63 | 15.14-126.14 |
| Mean | 10.17 | 0.30 | 4.54 | 3.96 | 281.80 | 37.50 | 12.61 | 75.20 |
| **Brackish-to-saline Transition Zone (M; n = 5)** | | | | | | | | |
| Range | 0.48-318.63 | 0-37.18 | 0-6.41 | 12.66-79.38 | 26.85-136.07 | 83.60-254.07 | 0.28-1.07 | 0.97-6.47 |
| Mean | 195.33 | 7.60 | 1.77 | 43.55 | 78.79 | 179.71 | 0.59 | 2.45 |
| **Hypersaline Lake (L; n = 39)** | | | | | | | | |
| Range | 2.27-122.65 | 0-16.15 | 0-81.25 | 19.55-53.3 | 169.18-256.2 | 2.04-51.39 | 3.84-15.06 | 4.60-11.05 |
| Mean | 20.29 | 2.73 | 13.97 | 25.62 | 229.21 | 32.41 | 9.36 | 9.31 |
